# Comparison of the biomarkers for targeted therapies in primary extra‐mammary and mammary Paget's disease

**DOI:** 10.1002/cam4.2820

**Published:** 2020-01-03

**Authors:** Zoran Gatalica, Semir Vranic, Božo Krušlin, Kelsey Poorman, Phillip Stafford, Denisa Kacerovska, Wijendra Senarathne, Elena Florento, Elma Contreras, Alexandra Leary, April Choi, Gino K. In

**Affiliations:** ^1^ Caris Life Sciences Phoenix AZ USA; ^2^ College of Medicine QU Health Qatar University Doha Qatar; ^3^ Ljudevit Jurak Department of Pathology and Cytology Clinical Hospital Center Sestre Milosrdnice Zagreb Croatia; ^4^ School of Medicine University of Zagreb Zagreb Croatia; ^5^ Medical Faculty in Pilsen Sikl's Department of Pathology Charles University in Prague Pilsen Czech Republic; ^6^ Bioptical Laboratory Pilsen Czech Republic; ^7^ Gustave Roussy Cancer Center INSERM U981 Paris France; ^8^ Norris Comprehensive Cancer Center University of Southern California Los Angeles CA USA

**Keywords:** extra‐mammary Paget's disease, immune therapy, molecular profiling, targeted therapy

## Abstract

**Background:**

Primary Extra‐mammary Paget's disease (EMPD) is a very rare cutaneous adenocarcinoma affecting anogenital or axillary regions. It is characterized by a prolonged course with recurrences and eventually distant metastatic spread for which no specific therapy is known.

**Methods:**

Eighteen EMPD (13 vulvar and five scrotal) and ten mammary Paget's disease (MPD) cases were comprehensively profiled for gene mutations, fusions and copy number alterations, and for therapy‐relevant protein biomarkers).

**Results:**

Mutations in *TP53* and *PIK3CA* were the most frequent in both cohorts: 7/15 and 5/15 in EMPD; 1/6 and 4/7 in MPD *HER2* gene amplification was detected in 4/18 EMPD (3 vulvar and 1 scrotal case) in contrast to MPD where it was detected in the majority (7/8) of cases. *TOP2A* gene amplification was seen in 2/12 EMPD and 1/6 MPD, respectively. Similarly, no difference in estrogen receptor expression was seen between the EMPD (4/15) and MPD (3/10). Androgen receptor was also expressed in the majority of both cohorts (12/16 EMPD) and (7/8 MPD).Here *ARv7* splice variant was detected in 1/7 EMPD and 1/4 MPD cases, respectively. PD‐L1 expression on immune cells was exclusively observed in three vulvar EMPD. In contrast to MPD, six EMPDs harbored a “high” tumor mutation burden (≥10 mutations/Mb). All tested cases from both cohorts were MSI stable.

**Conclusions:**

EMPD shares some targetable biomarkers with its mammary counterpart (steroid receptors, PIK3CA signaling pathways, *TOP2A* amplification). HER2 positivity is notably lower in EMPD while biomarkers to immune checkpoint inhibitors (high TMB and PD‐L1) were observed in some EMPD. Given that no consistent molecular alteration characterizes EMPD, comprehensive theranostic profiling is required to identify individual patients with targetable molecular alterations.

## Graphical abstract

Comprehensive molecular profiling of primary Extra‐mammary Paget's Disease (EMPD) indicates that individually profiled cases harbor potentially targetable molecular alterations. EMPD shares some but not all molecular features with its mammary counterpart. Potential therapeutic targets in EMPD include steroid receptors (ER and AR), HER2, and PIK3CA signaling pathways, TOP2A amplification, and immune checkpoints (PD‐L1).

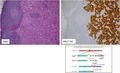

## 1 INTRODUCTION

Primary extra‐mammary Paget's disease (EMPD) is a very rare, cutaneous adenocarcinoma of uncertain etiology commonly affecting anogenital or axillary regions. The Surveillance, Epidemiology and End Results (SEER) Registry reported an incidence of ~2200 cases in the United States over 40 years.^1^ In contrast to the more common mammary Paget's disease, which is the manifestation of intra‐epidermal dissemination of an underlying invasive or in situ breast carcinoma, or secondary extra‐mammary pagetoid spread of adenocarcinomas from various internal organs, primary cutaneous EMPD lacks an underlying malignancy.^2^ Primary EMPD is a slowly progressive disease and is usually diagnosed while at the in situ (intra‐epidermal) stage. Following dermal invasion, it metastasizes to regional lymph nodes and potentially other distant sites.^3^ The postsurgical (local) recurrence rate in EMPD is 20%‐40% and metastatic EMPD has a poor survival rate.^3^

Due to its rarity, standard systemic treatment protocol for EMPD is currently not established.^3^, ^4^, ^5^

In this study, we compared comprehensive molecular‐genetic profiles of a cohort of primary EMPD to primary mammary Paget's disease (MPD) to detect common and distinguishing tumor characteristics, providing additional supportive evidence for optimal therapy approaches.^6^

## 2 MATERIALS AND METHODS

### 2.1 Samples

Cases of primary EMPDs [intra‐epidermal and invasive (advanced or metastatic) stages] were retrospectively analyzed from the tumor samples submitted for molecular profiling. The histologic diagnosis and accompanying diagnostic immunohistochemical workup performed at the referring pathology laboratories were reviewed in all cases by a board‐certified pathologist. Cases of secondary EMPD (intra‐epidermal spread from an underlying carcinoma, e.g., colon, rectum, anus, prostate) were excluded from the study. A cohort of mammary Paget's disease (MPD) of the breast was used for comparison.

All test assays were performed at CLIA/CAP/ISO15189/NYSDOH certified clinical laboratory. Additional molecular assays were performed on de‐identified remnant specimens as required. The study was deemed exempt from IRB approval and consent requirements were waived in compliance with 45 CFR 46.101(b), as all remnant tissues and biomarker data were analyzed with no associated identifiers.

### 2.2 Immunohistochemistry (IHC)

PD‐L1 expression was evaluated in the tumor (TC) and immune cells (IC) using SP142 antibody (Ventana). Any PD‐L1 expression was considered positive if either TC or IC exhibited staining. AR (clone 441, Leica Biosystems, Buffalo Grove, IL), ER (SP1 clone, Ventana, Tucson, AZ) and PR (1E2, Ventana, Tucson, AZ) were analyzed using a ≥10% threshold for nuclear positivity. HER2 (4B5 clone, Ventana) was considered positive if >10% cancer cells showed complete, circumferential (3+) expression or exhibited *HER2* gene amplification (see below). Nine cases (five vulvar and four scrotal) of EMPD and four MPD were explored for the expression of the splice variant of AR (ARv7) using immunohistochemistry (EPR15656, Abcam). Three EMPD cases were tested for mismatch repair proteins: MLH1 (Clone M1, Ventana), MSH2 (Clone G219‐1129, Ventana), MSH6 (Clone 44, Cell Marque) and PMS2 (Clone EPR3947, Cell Marque). Topoisomerase 2α (Clone 3F6, Leica) expression was considered positive if cancer cells exhibited nuclear positivity in ≥10%.^7^

### 2.3 Chromogenic in situ hybridization (CISH)

Chromogenic in situ hybridization (CISH) was used for evaluation of the *HER2* (*HER2/CEP17* [chromosome 17 centromere] probe) and *TOPO2A* (*TOP2/CEP17* probe) (Abbott Molecular/Vysis, Abbott Park, IL). *HER2/CEP17* and *TOPO2A/CEP17* ratios ≥2.0 were considered amplified.^7^

### 2.4 Next‐generation sequencing (NGS)

All specimens were profiled using massively parallel sequencing (NGS). The NGS assay encompasses a 592‐gene panel that utilizes SureSelect XT biotinylated RNA probes to capture DNA fragments from exons of 592 genes (Agilent).^8^

We also assessed copy number alterations of 442 genes (CNA) with the NGS panel. CNAs were calculated by comparing the depth of sequencing of genomic loci to a diploid control as well as the known performance of these genomic loci over several hundred historical cases. Gains ≥6 copies were considered amplified.^8^, ^9^

**Figure 1:**
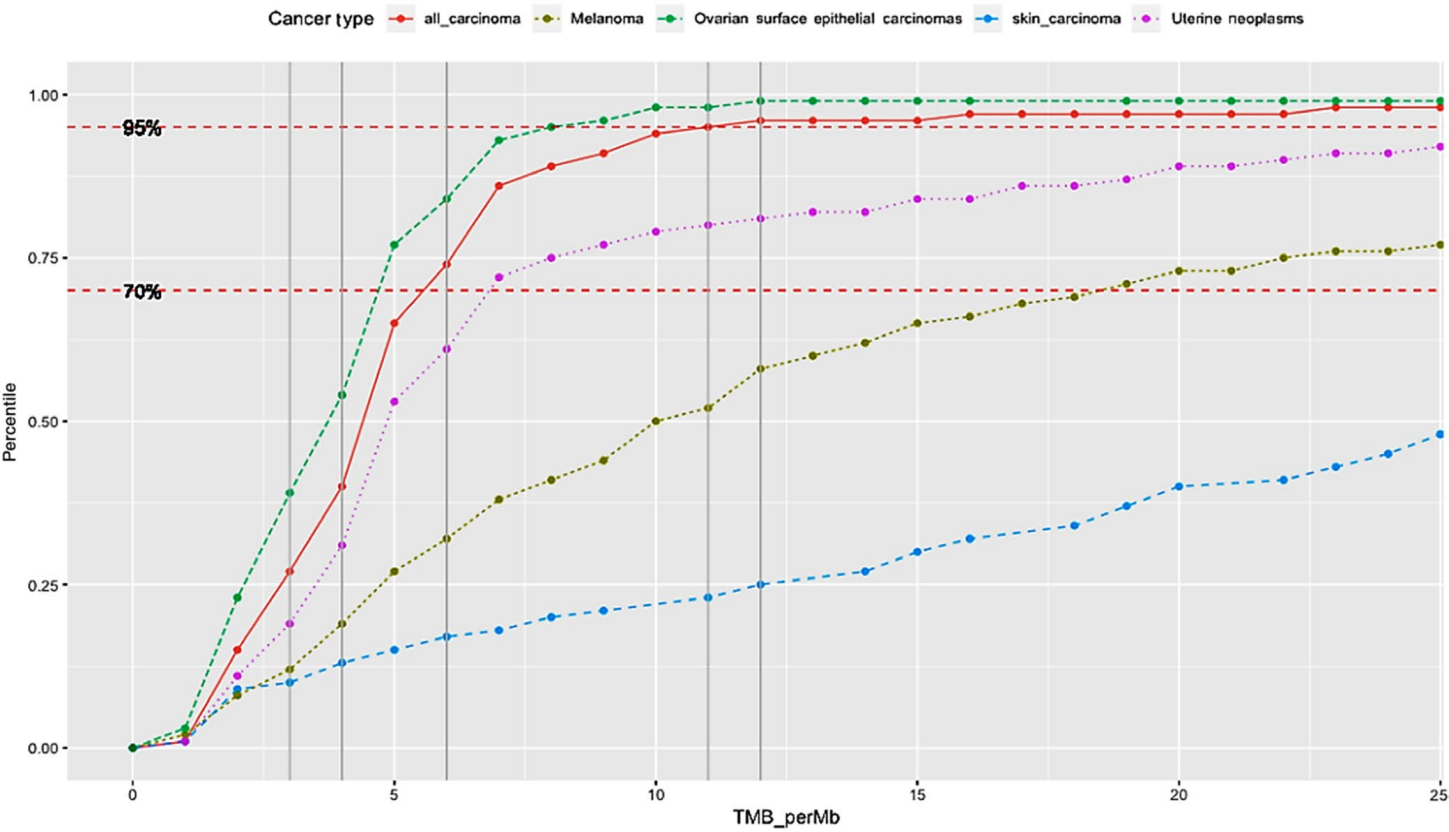
Plot of tumor mutational burden (TMB) (*x*‐axis) vs percentile (*y*‐axis) for cancer subtypes. Gray vertical lines reflect the TMB values of EMPD cases on *x*‐axis and where they intersect with curves for different cancer types is where they fall in terms of percentile (*y*) for the specific groups. Red dots represent all carcinomas; dark green dots—melanomas; blue dots—skin carcinomas, green dots—ovarian surface epithelial carcinomas and purple dots—uterine neoplasms

Genome‐wide mutational signatures were derived by analyzing the NGS data as previously reported. After excluding variants included in dbSNP 137 (National Center for Biotechnology Information, National Institutes of Health) and 1000 genomes (IGSR, International Genome Sample Resource, EMBL‐EBI) as presumed germline variants from a VCF, the number of missense mutations in the VCF is counted and divided by 1.4 Mb, which is the total size of target CDSs, to calculate tumor mutational burden (TMB). In order to allow comparison of mutational burden across tumor types, we converted TMB value to percentile, a nonparametric rank that removes the impact of deviations from normality in the underlying TMB distribution. The percentile is calculated using 500 patient cases that represent the range of TMB values for that cancer type. When there are fewer than 500 cases for a given tumor type, we consolidate multiple classifications. Figure 1 shows several representative curves for percentile vs. TMB for two different combined cancer classifications (“All Carcinoma” and “Skin Carcinomas”), contrasting to melanoma and gynecological cancers (ovarian epithelial and uterine). In Figure 1, we also include a line at the point where our EMPD patients TMB values would fall.

Microsatellite instability (MSI) was calculated by direct analysis of short tandem repeats in the target regions of sequenced genes. The count only included alterations that resulted in increases or decreases in the number of repeats. MSI‐H was defined as ≥46 altered microsatellite loci. The threshold was established by comparing NGS with the PCR‐based microsatellite fragments analysis results from ~2100 cases.^8^, ^9^, ^10^

ArcherDx FusionPlex Assay (ArcherDX) was used for detection of gene fusions including *ARv7* variant transcript. In total, 54 gene targets were analyzed in seven EMPD (five vulvar and two scrotal) and four MPD cases.^8^

## 3 RESULTS

### 3.1 Clinicopathologic characteristics of the cohorts

The study included 18 cases of primary EMPD and 10 cases of MPD (Table 1). Among the EMPD cases, there were 13 patients with vulvar involvement and 5 cases from scrotum/perianal region. Most cases represented invasive and/or advanced/metastatic EMPD (10/13 vulvar and 5/5 scrotal EMPD) (Table 1). All patients were clinically investigated; no underlying malignancy was found, thereby ruling out the possibility of secondary EMPD. All MPD were localized to the breast, half (5) of them had underlying ductal carcinoma in situ (DCIS) or invasive mammary carcinoma (Table 1).

**Table 1:** Patients’ demographics and sample sites from the two cohorts

Tumor type (number)	Extra‐mammary Paget's disease (n = 18)	Mammary Paget's disease (n = 10)
Age: Mean and range	61 y (49‐82 y) (vulva) 73.5 y (69‐79 y) (scrotum)	62 y (37‐76 y, all females)
Sample site	Vulva (n = 13) (10/13 advanced or metastatic) Scrotum (n = 5) (all metastatic)	All primary (n = 10) 5 with underlying DCIS or invasive mammary carcinoma

### 3.2 Steroid receptors profile

Two out of eleven evaluated cases of vulvar EMPD were positive for ER. In contrast, AR was positive in the majority of the cases (9/11). Among the EMPD of scrotum, ER was positive above the 10% threshold in 2/4 cases, while AR was positive in 3/5. Similarly, ER expression was observed in 3/10 MPD while AR was positive in the majority of the cases (7/8) (Table 2).

**Table 2:** Overview of the identified biomarkers in the 18 cases of extra‐mammary Paget's disease

Case	Type	Location (biopsy site)	Age	Steroid receptors	HER2 status	PD‐L1	TML	MSI status	Mutational profile*	Other findings
Extramammary Paget's Disease (vulva) (n = 13)										
#1	Advanced	Vulva	49	ER−/AR−	Negative	n/a	n/a	n/a	n/a	None
#2	Primary	Vulva	82	ER+/AR+	Negative	n/a	n/a	n/a	n/a	Topo2α positive
#3	Metastatic	Liver (primary site: vulva)	64	ER−/AR−	Positive (amplified)	Negative	n/a	n/a	w.t.	none
#4	Metastatic	Lymph node (primary site: Vulva)	51	ER−/AR+	Negative (not amplified)	Negative	6	Stable	w.t.	none
#5	Metastatic	Lymph node (primary site: Vulva)	43	ER−/AR+	Negative (not amplified)	Negative	12	Stable	*PIK3CA* (H1047R) *BCOR*	none
#6	Primary	Vulva	64	ER−/AR+	Positive but not amplified	Negative	n/a	n/a	*TP53* (R248Q)	Topo2α positive
#7	Advanced	Vulva	70	n/a ARv7 (−)	Negative (not amplified)	Negative	11	Stable	*TP53* (R248Q), *PIK3CA* amplified	Topo2α positive
#8	Advanced	Vulva	55	ER−/AR+/ ARv7 (+)	Positive (amplified)	Positive in IC	12	Stable	*TP53* (Q192X), *SETD2* (S845X and S1390x)	*TOP2A* amplified
#9	Advanced	Vulva	67	n/a ARv7 (−)	Positive (amplified)	Negative	6	Stable	*TP53, PIK3CA, BRCA1, RB1, MUTYH*	Topo2α positive
#10	Advanced	Vulva	n/a	ER−/AR+ ARv7 (−)	Negative (not amplified)	Positive IC at the tumor interface	3	Stable	*PIK3CA* (R88Q)	*TOP2A* amplified
#11	Advanced	Vulva	62	ER+/AR+	Negative (not amplified)	Negative	n/a	n/a	n/a	Topo2α positive
#12	Advanced	Vulva	n/a	ER−/AR+ ARv7 (−)	Negative (not amplified)	Positive IC	6	Stable	*SETD2* (S2148X)	Topo2α positive
#13	Primary	Vulva	66	ER−/AR+	Negative (not amplified)	Negative	n/a	n/a	*SETD2* (unclassified)	Topo2α positive
Extra‐mammary Paget's Disease (scrotum/perineum/perianal) (n = 5)										
#1	Advanced	Scrotum	79	ER+/AR+	Negative (not amplified)	n/a	n/a	n/a	*PIK3CA (E545K)*	Topo2α positive
#2	Recurrent	Buttock/ perianal	69	ER+/AR+ ARv7 (−)	Positive (amplified)	Negative	11	Stable	*TP53* (R280K), *MUTYH* (G393D)	Topo2α positive
#3	Metastatic	Axillary lymph node (scrotum)	72	ER−/AR−	Negative (not amplified)	Negative	11	Stable	*TP53 (P152T)*	None
#4	Metastatic	Perineum	75	n/a	Negative (not amplified)	Negative	11	Stable	*TP53 (c.376‐9_382del16), PIK3CA (H1047R),*	Topo2α positive
#5	Metastatic	Axillary lymph node (scrotum)	76	ER−/AR+ ARv7 (−)	Negative (not amplified)	Negative	4	Stable	None	None

* Only pathogenic mutations are listed.

**Figure 2:**
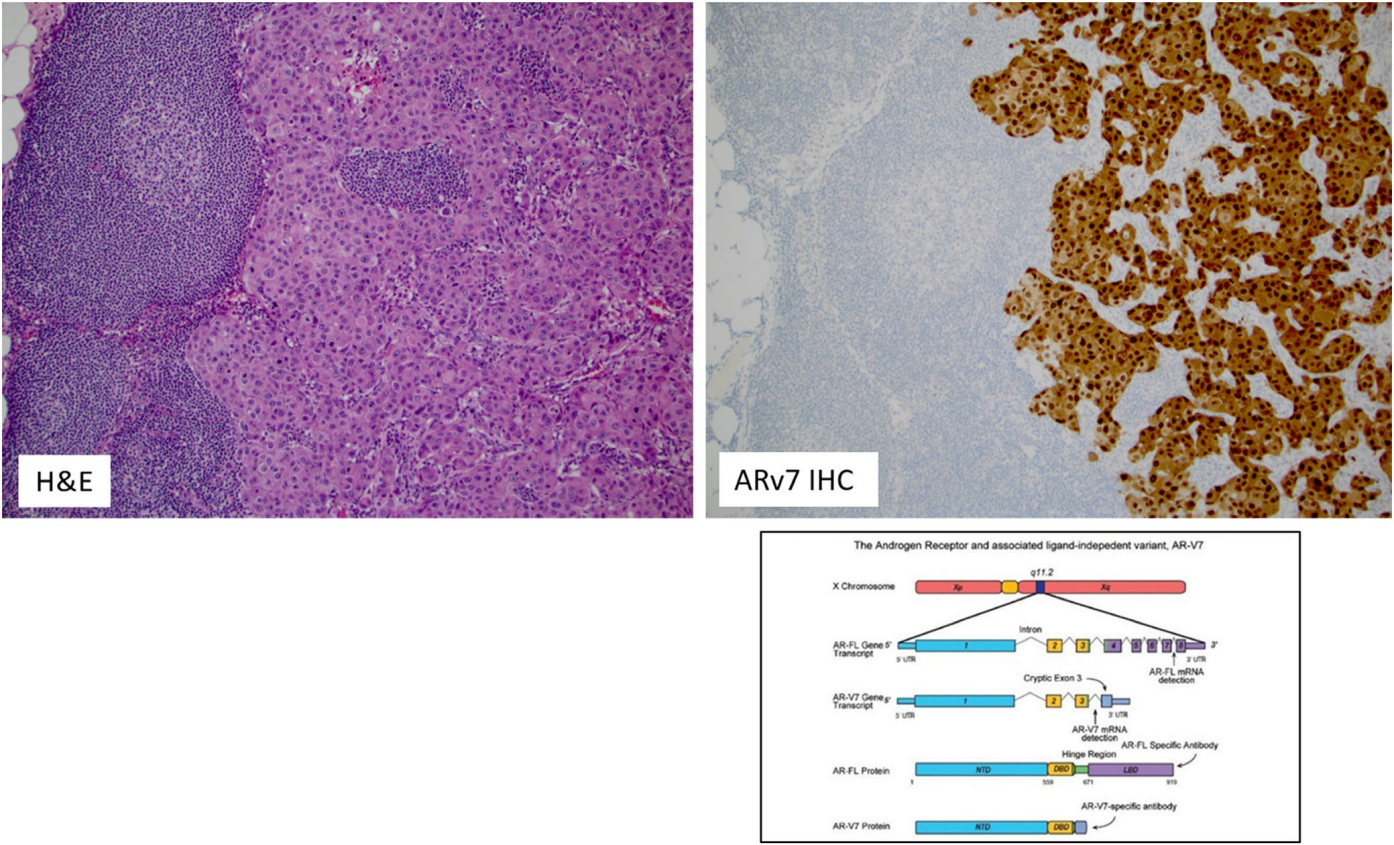
Hematoxylin and Eosin (H&E) slide of the metastatic (lymph node) case of EMPD (left image) with diffuse and strong positivity for ARv7 by immunohistochemistry (right image). The figure in the lower right corner depicts the structure of the Androgen receptor (*AR*) gene and its associated ligand‐independent variant *ARv7*. ARv7 can be reliably detected by immunohistochemistry (IHC) as shown in the upper right image. Transcriptome structure for AR gene and its splice variants modified from Luo J. Asian J Androl 2016;18: 580–585

The *ARv7* variant transcript was detected by RNA‐sequencing in 1/5 of vulvar EMPD and 1/4 MPD. This observation was further confirmed by a positive (nuclear) staining of ARv7 by IHC in a case of EMPD (Figure 2). No scrotal cases expressed the *ARv7* variant (0/2)*.*

### 3.3 HER2 and TOP2A status

**Figure 3:**
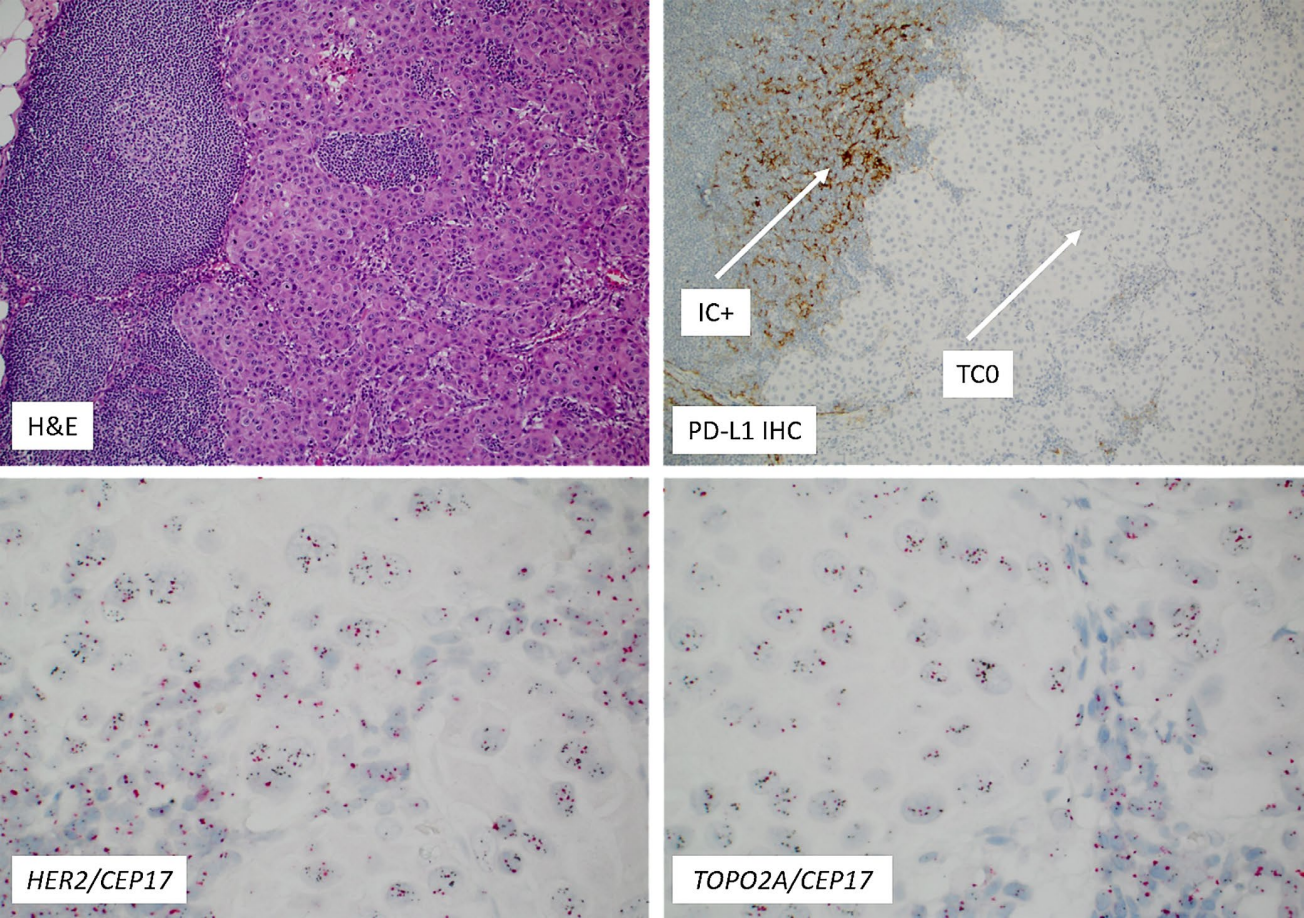
The same case provided in Figure 2 with a PD‐L1 positivity in the inflammatory (immune cells, IC; upper right image, white arrow); Please note the absence of PD‐L1 expression in tumor cells (TC; white arrow); Lower two images show *HER2* (left) and *TOP2A* gene co‐amplifications (right image) (Chromogenic in situ hybridization). The black spots on the images reflect *HER2* and *TOP2A* gene copy numbers, respectively, while the red spots indicate the *CEP17* region

*ERBB2/HER2* gene amplification by CISH or CNA/NGS was detected in 3/13 vulvar and 1/5 scrotal cases (Figure 3); among these all four amplified cases also showed positive IHC HER2 expression (Table 1). In addition, one case of vulvar EMPD showed positive protein expression by IHC without gene amplification. None of the cases harbored pathogenic *ERBB2* (*HER2*) gene mutations; however, the single HER2 positive scrotal case did harbor a variant of unknown significance in the *ERBB2* gene (E580K and E619K). In comparison, a notably higher HER2 overexpression was observed in the small cohort of MPD (7/8).

*TOP2A* gene amplification was observed in two vulvar EMPD (one case had also co‐amplification of *HER2,* Figure 3) and 1/6 MPD; All amplified cases exhibited Topo2α protein overexpression by IHC. Additionally, a total of nine vulvar and three scrotal cases showed Topo2α protein overexpression by IHC (Table 2). Two nonamplified MPD were also positive for Topo2α by IHC.

### 3.4 I‐O relevant biomarkers

None of the EMPD cases exhibited aberrant expression of PD‐L1 in cancer cells (TC, 0/14). Immune cell (IC) expression of PD‐L1 was observed in three vulvar EMPD of which two cases had an intense PD‐L1 staining of immune cells, especially at the tumor‐stromal interface (Figure 3).

TMB in vulvar cases varied between 3 and 12 mutations/Mb; three cases had 10 or more mutations/Mb. TMB was available for four scrotal cases, 3 of which had 11 mutations/Mb, while the fourth case showed 4 mutations/Mb. We placed markers on Figure 1 to allow a comparison of the TMB percentiles for each of the listed cancer cohorts against the TMB values for EMPD patients.

All tested EMPD, either vulvar (n = 7) or scrotal (n = 4), were microsatellite stable. Three cases were additionally tested by immunohistochemistry for mismatch repair proteins and all cases retained normal expression.

All predictive I‐O biomarkers in MPD were negative: PD‐L1 expression in TC (0/10), high TMB (0/1) and MSI status (5/5 stable).

### 3.5 Genomic alterations

Pathogenic mutations in *TP53* and *PIK3CA* were the most common genomic alterations detected in vulvar EMPD (4/10 and 3/10, respectively). *PIK3CA* mutations were found at well‐described oncogenic loci (https://cancer.sanger.ac.uk/cosmic/). One case also harbored a *PIK3CA* gene amplification by NGS, suggesting amplification as an alternate mechanism of pathway activation. Pathogenic *SETD2* gene mutations were present in two cases (20%) while other gene mutations (*BRCA1, RB1, BCOR*, and *MUTYH*) were rare and affected only one case each. Interestingly, one case harbored two separate nonsense mutations in *SETD2*, suggesting complete gene activation. In scrotal EMPD, *TP53* and *PIK3CA* mutations were present in n = 3 and 2 of 5 cases, respectively. In one scrotal EMPD, a pathogenic *MUTYH* gene mutation (G393D) was found representing a germline variant (MUTYH‐associated polyposis is an autosomal recessive polyposis syndrome caused by bi‐allelic pathogenic germline variants in the MUTYH gene.^11^ No other CNV or gene fusions were detected in any of the tested cases.

*PIK3CA* mutations were also common in MPD (4/7) while *TP53* mutations were observed in 1/6 tested cases. Notably, three out of four *PIK3CA* mutated cases were ER+. Although rare, several genomic alterations were exclusively detected in MPD: *CHEK2* and *CDK12* gene mutations (1 case each) and *MLLT6* (2/5) and *MDM2* gene amplifications (1/5) (Table 3).

**Table 3:** Comparative overview of the molecular features of the extra‐mammary‐ and mammary Paget's disease

Biomarker	Extra‐mammary Paget's disease (positive/total)	Mammary Paget's disease (positive/total)
Steroid receptors		
Estrogen receptor (ER)	4/15	3/10
Androgen receptor (AR)	12/16	7/8
*ARv7*	1/7	1/4
Genomic alterations		
*HER2 (ERBB2)* (amp.)	4/18	7/8
*TOP2A* (amp.)	2/12	1/6
*PIK3CA*	5/15	4/7
*TP53*	7/15	1/6
*SETD2*	2/15	0/5
*BRCA1*	1/15	0/5
*RB1*	1/15	0/7
*MLLT6*	0/15	2/5
*CHEK2*	0/15	1/5
*CDK12*	0/15	1/5
*MDM2* (amp.)	0/12	1/5
I‐O biomarkers		
PD‐L1 expression	3/14 (vulva, IC+)	0/10
High tumor mutational burden*	6/11	0/1
Microsatellite instability (MSI)	Stable (n = 11)	Stable (n = 5)

Abbreviations: Amp., Amplification by In situ hybridization; IC, Inflammatory (immune) cells; I‐O, Immuno‐oncology.

* Threshold for TMB‐high was set at ≥ 10

## 4 DISCUSSION

In contrast to mammary Paget's disease, primary EMPD is a rare and slowly progressive skin adenocarcinoma. In its superficial phase and if surgically resectable, it is associated with a good prognosis.^12^ Once it invades dermis and deep structures, the risk of lymph node and distant metastases significantly increases.^13^, ^14^ Due to its rarity, no systemic, comprehensive evaluation of targeted therapies has been performed. Selected cases of EMPD (with amplified *ERBB2/HER2* gene) have been successfully treated with trastuzumab alone or in combinations with chemotherapy.^4^, ^15^, ^16^, ^17^, ^18^, ^19^, ^20^, ^21^ In contrast to MPD, we found overexpression and *HER2* gene amplification in a minority of cases, and a co‐amplification with *TOPO2* in a single case of vulvar disease. Amplification of *TOPO2* gene and expression of Topo2 protein in several cases of EMPD is a novel finding in our cohort. TOPO2α is the target for anthracyclins^22^ chemotherapy and this biomarker was frequently altered in our study. Topo2α protein is commonly expressed across human cancers while *TOP2A* gene amplification is rare, with only few cancer types (gallbladder and gastroesophageal/esophageal carcinomas) exhibiting *TOP2A* amplification in >10% of the cases.^23^ Based on the Topo2α/*TOP2A* status alone, a substantial proportion of the patients with advanced/metastatic EMPD may benefit from anthracycline‐based chemotherapy.

Although early studies reported the lack of ER expression in EMPD,^24^, ^25^, ^26^ we detected ER positivity in a small number of EMPD. Garganese et al recently reported ER positivity in 70% of vulvar EMPD.^27^ Importantly, this may represent a potential therapeutic target as Iijima et al reported a successful treatment of ER + scrotal EMPD with tamoxifen.^28^ In addition, Wachter et al recently reported ER expression in 27% of MPD.^29^

Similar to MPD from our cohort, androgen receptor (AR) over‐expression is commonly seen in EMPD (54%‐90%).^24^, ^27^, ^30^ AR protein expression has also been shown to correlate with the invasiveness in EMPD.^3^ A successful treatment with combined androgen blockade (bicalutamide/targeted anti‐AR/) and leuprolide acetate (LH‐RH analogue) has been published in a single case report.^31^ Our study further confirms earlier observations of frequent AR expression.^24^, ^32^ We also report for the first time, the presence of the ARv7 transcript, a pathogenic splice variant of AR in single cases of EMPD and MPD. This variant of AR lacks the ligand‐binding domain (a target of anti‐AR drugs such as enzalutamide and abiraterone), but retains its constitutive activity.^33^ Consequently, ARv7 causes resistance to anti‐AR drugs as confirmed in patients with prostate cancer.^33^ Given the common AR expression and the lack of ARv7 in the majority of AR + EMPD, these patients may be amenable for the trials with anti‐AR drugs.^31^, ^34^

Immunotherapy with immune checkpoint inhibitors has become the cornerstone of treatment for several advanced cancers such as NSCLC, melanoma, urothelial carcinoma, head and neck carcinomas, and triple negative breast carcinomas. For some of these cancers, approval is based on companion diagnostic testing for the expression of PD‐L1 in either cancer cells (TC), immune cells (IC; mononuclear cells, excluding polymorphonuclear leukocytes), or both (eg combined positive score), depending on cancer types. In our study, none of the cases exhibited aberrant PD‐L1 expression on neoplastic cells (TC 0) and three EMPD cases had intense PD‐L1 positive immune cells infiltrate. Recently, Mauzo et al demonstrated low (3/21) PD‐L1 expression on cancer cells while immune cells (IC) expressed PD‐L1 in 15/21 of the EMPDs. In addition, PD‐L1 expression in ICs was significantly lower in patients with HER2‐positive compared with HER2‐negative EMPD cancers.^35^ Duverger et al reported positive expression of PD‐L1 on cancer cells in n = 4/7 invasive EMPD,^36^ while Karpathiou et al reported that EMPD are characterized by the intense lymphocytic response but without PD‐L1 expression on either cancer or immune cells.^37^ A single patient in our cohort was included in a basket trial with Pembrolizumab without clinical benefit and a later tumor sample analysis revealed no PD‐L1 positivity, but *HER2* amplification was detected and Herceptin therapy was initiated (data not shown). Interestingly, one of the *TOP2A*‐amplified cases also exhibited PD‐L1 positivity in IC giving a potential for combination chemotherapy with immune checkpoint inhibitors.^6^

Additional immune‐oncology markers such as high TMB are under investigation as part of late phase clinical trials.^38^, ^39^ However, it remains to be seen whether immune checkpoint inhibitors will have efficacy for the treatment of EMPD. We observed some patients with high TMB and the presence of PD‐L1 in IC (TIL) in our study of EMPD, indicating that these patients may also benefit from treatment with immune checkpoint inhibitors (ClinicalTrials.gov Identifier: NCT02834013). We suggest there is value in using a relative ranking for TMB. One simple method is converting TMB to rank (percentile) using a cohort of patients with the same cancer diagnosis. TMB percentile may be a more meaningful way to assess the likely impact global mutations have on therapy options.

MSI is an additional measure of genome instability, leading to possible therapies. Notably, all EMPD cases from our cohort were MSI stable (by NGS and IHC), which is in stark contrast to the study of Kang et al that showed that 8/20 EMPD harbored germline mutations of mismatch repair genes (MMR), five of which were MSI‐H.^40^ However, both studies have rather limited number of cases studied, and vigilance about MSI status in EMPD is warranted. In contrast to EMPD, MPD cases were entirely devoid of PD‐L1 expression; all cases were also MSI stable and lacked high TMB, making MPD patients unlikely to respond to immune checkpoint inhibitors. These results are in line with previous data.^37^

Next‐generation sequencing from this cohort is in line with previous studies.^41^, ^42^, ^43^ In contrast to Kiniwa et al who reported mutations in *ERBB2* gene,^43^ we found only *ERBB2* amplification but no pathogenic *ERBB2* mutations in any of our cases. The presence of mutations within the PIK3CA/AKT pathway indicates a potential for targeted therapeutic trials with newer generation of PIK3CA inhibitors, either alone or in combination with other therapies.^44^, ^45^ Recently, a PIK3CA inhibitor was FDA approved for the treatment of ER‐positive/HER2‐negative breast cancers harboring known pathogenic *PIK3CA* mutations in combination with anti‐ER therapy (fulvestrant) (FDA site, accessed October 12, 2019). One of the scrotal EMPD from our cohort with *PIK3CA* mutation was ER‐positive. Notably, three out of four PIK3CA‐mutated MPD cases were ER‐positive and are potential candidates for the treatment with PIK3CA inhibitors. Similarly, AR+/PIK3CA mutated cases might benefit from the combined treatment with anti‐AR and PIK3CA inhibitors as recently shown in phase IB/II clinical trial with triple‐negative breast cancer.^45^

We report here for the first time two EMPD cases with *SETD2* gene mutations, which have not been previously described in this cancer. Inactivating *SETD2* mutations are common in clear cell renal cell carcinomas, but have been described at low frequencies across additional tumor types. *SETD2* is responsible for trimethylation of lysine 36 of Histone H3 (H3K36),^46^ which is involved in various cellular processes. *SETD2*‐mutant cancers have showed a substantial decrease in global H3K36me3 levels.^47^ Additionally, the loss of function of *SETD2* has been shown to sensitize tumors to PI3KB inhibitors^48^ and is under investigation as a target for other agents in phase I and II clinical trials, making it a novel potential therapeutic target in this disease.

We conclude that EMPD shares some targetable biomarkers with its mammary counterpart (steroid receptors, PIK3CA pathways, *TOP2A* amplification). HER2 positivity is notably lower in EMPD while expression of biomarkers to immune checkpoints (high TMB and IC PD‐L1) was observed in some EMPD. In selected EMPD cases with more than one targetable biomarker, a combination therapy may be a part of the precision medicine approach. Therefore, comprehensive case‐by‐case analysis is required to maximize benefits of the targeted therapeutic for patients with this rare disease.

## CONFLICT OF INTEREST

Zoran Gatalica, Kelsey Poorman, Phillip Stafford, Wijendra Senarathne, Elma Contreras, and Elena Florento are employees of Caris Life Sciences. Alexandra Leary serves on the advisory boards of AstraZeneca, Clovis, Tesaro, GlaxoSmithKline, Merck & Co., Merck Serono, GamaMabs Pharma SA, Seattle Genetics, BIOCAD, and Ability Pharma. She has been receiving travel funds from AstraZeneca, Roche and Tesaro. Investigator on trials funded by: Pfizer, Merus, Roche, Agenus, Merck & Co, Clovis, AstraZeneca, and Ability Pharma. Gino K. In serves on advisory boards of Bristol‐Myers Squibb, Castle and Novartis. He has been a speaker for Merck and investigators for trials funded by Roche and Idera Pharmaceuticals. Semir Vranic had received honoraria from Caris Life Sciences. The other authors declare no conflict of interest.

## AUTHOR CONTRIBUTIONS

Zoran Gatalica: Conceptualization, methodology, data curation, formal analysis, validation, supervision, and writing–review and editing; Semir Vranic: Data curation, formal analysis, supervision, writing‐review and editing; Božo Krušlin: Formal analysis, writing‐review and editing; Kelsey Poorman: Formal analysis, validation, writing‐review; Phillip Stafford: Methodology, formal analysis, validation, writing‐review; Denisa Kacerovska: Formal analysis, validation, writing‐review; Wijendra Senarathne: Formal analysis, validation, writing‐review; Elena Florento: Data curation, formal analysis, writing‐review; Elma Contreras: Data curation, formal analysis, writing‐review; Alexandra Leary: Formal analysis, validation, writing‐review; April Choi: Data curation, writing‐review; Gino K. In: Supervision, and writing–review and editing.
